# ST2 Deficiency Does Not Impair Type 2 Immune Responses during Chronic Filarial Infection but Leads to an Increased Microfilaremia Due to an Impaired Splenic Microfilarial Clearance

**DOI:** 10.1371/journal.pone.0093072

**Published:** 2014-03-24

**Authors:** Jesuthas Ajendra, Sabine Specht, Anna-Lena Neumann, Fabian Gondorf, David Schmidt, Katrin Gentil, Wolfgang H. Hoffmann, Mark J. Taylor, Achim Hoerauf, Marc P. Hübner

**Affiliations:** 1 Institute for Medical Microbiology, Immunology and Parasitology, University Hospital Bonn, Bonn, Germany; 2 Institute of Tropical Medicine, University of Tuebingen, Tuebingen, Germany; 3 Filariasis Research Laboratory, Department of Parasitology, Liverpool School of Tropical Medicine, Liverpool, United Kingdom; INRS - Institut Armand Frappier, Canada

## Abstract

**Background:**

Interactions of the Th2 cytokine IL-33 with its receptor ST2 lead to amplified Type 2 immune responses. As Type 2 immune responses are known to mediate protection against helminth infections we hypothesized that the lack of ST2 would lead to an increased susceptibility to filarial infections.

**Methodology/Principal Finding:**

ST2 deficient and immunocompetent BALB/c mice were infected with the filarial nematode *Litomosoides sigmodontis*. At different time points after infection mice were analyzed for worm burden and their immune responses were examined within the thoracic cavity, the site of infection, and systemically using spleen cells and plasma. Absence of ST2 led to significantly increased levels of peripheral blood microfilariae, the filarial progeny, whereas *L. sigmodontis* adult worm burden was not affected. Development of local and systemic Type 2 immune responses were not impaired in ST2 deficient mice after the onset of microfilaremia, but *L. sigmodontis* infected ST2-ko mice had significantly reduced total numbers of cells within the thoracic cavity and spleen compared to infected immunocompetent controls. Pronounced microfilaremia in ST2-ko mice did not result from an increased microfilariae release by adult female worms, but an impaired splenic clearance of microfilariae.

**Conclusions/Significance:**

Our findings suggest that the absence of ST2 does not impair the establishment of adult *L. sigmodontis* worms, but is important for the splenic clearance of microfilariae from peripheral blood. Thus, ST2 interactions may be important for therapies that intend to block the transmission of filarial disease.

## Introduction

ST2 is a member of the IL-1 receptor family that was recently described as the receptor for the Th2 cytokine IL-33 [1]. Despite its structural similarity with the IL-1 receptor family, ST2 does not bind to either IL-1α or IL-1β. IL-33 is released from various tissues and organs [1] including epithelial barrier tissues, lymphoid organs, brain and inflamed tissues. In addition, mast cells, type-2 pneumocytes, alveolar macrophages, and inflammatory dendritic cells have been described as cellular sources [2], [3], [4], [5]. ST2 expression has been described on Th2 cells [6], mast cells [7], eosinophils, basophils, neutrophils [8], and type 2 innate lymphoid cells (ILC2s) [9]. Administration of IL-33 amplifies Type 2 immune responses by inducing the expression of the Th2 cytokines IL-4, IL-5 and IL-13; driving eosinophilia, splenomegaly and serum IgE [1]; stimulating the differentiation of alternatively activated macrophages [10]; and activating basophils [8].

In recent years several groups have demonstrated that Type 2 immune responses protect against helminth infections, although Type 2 independent protective mechanisms do exist. With regard to filarial infections, mice that are deficient for either IL-4 or IL-5 harbor increased numbers of first stage larvae, termed microfilariae, the offspring of filarial adults, and a higher adult worm burden has also been noted in the absence of IL-5 [11]. Furthermore, mice that are deficient for the eosinophil products major basic protein 1 (MBP) and eosinophil peroxidase (EPO) were shown to have increased adult worm burden despite higher eosinophilia [12].

Thus, the interaction of IL-33 and its receptor ST2 may impact the ensuing protective immune reactions towards helminths by supporting the development of a Type 2 immune response. Indeed, the absence of ST2 has already been associated with impaired Th2 immune responses following immunization with *Schistosoma mansoni* eggs and moreover, the abrogation of granuloma formation [13]. A possible protective role of IL-33 in helminth infections was suggested by a study from Humphreys *et al.* that showed that the administration of IL-33 during an early phase of infection with *Trichinella spiralis* induced a protective Type 2 immune response that expelled the parasite [14]. However, administration of IL-33 at a chronic stage of infection did not improve resistance to *T. spiralis* [14]. An ST2-dependent Th2 immune response during *T. spiralis* infection was also shown by Scalfone *et al.* [15]. In their study ST2-ko mice expelled adult worms in a manner reflected in wildtype (WT) mice, but had significantly increased larvae numbers in the muscle, suggesting an ST2 dependent effect on worm fecundity or larval migration [15].

Given that the interaction of IL-33 and ST2 induces a Type 2 immune response, the focus of this study was to investigate whether the loss of the ST2 receptor impairs the development of a filarial nematode induced Type 2 immune response, thus resulting in an increased worm burden. To investigate this hypothesis we used the filarial nematode *Litomosoides sigmodontis*, a well-established murine model for human filariasis [16], [17]. Natural infections with *L. sigmodontis* L3 larvae in susceptible BALB/c mice result in the development of patent infections, the release of microfilariae which enables transmission of the infection via blood feeding vectors. Using ST2 knockout BALB/c mice and corresponding WT controls, we show here that lack of signaling via ST2 leads to increased numbers of microfilariae due to an impaired splenic clearance.

## Materials and Methods

### Ethics Statement

Animal housing conditions and the procedures used in this work were performed according to the European Union animal welfare guidelines. All protocols were approved by the Landesamt für Natur, Umwelt und Verbraucherschutz, Cologne, Germany (AZ 87-51.04.2011.A025/01) and by the Regierungspräsidium Tübingen, Germany (T1/96).

### Mice and parasites

ST-2 deficient mice and WT controls (kindly provided by Professor Andrew McKenzie, University of Cambridge, UK) were housed and bred at the animal facility of the Institute of Medical Microbiology, Immunology and Parasitology, University Hospital Bonn, and had access to food and water *ad libitum*. Age and sex-matched mice were infected at 6–8 weeks of age with *L. sigmodontis* via natural infection with the intermediate host as described before [11]. To ensure comparable infections of both groups, mice from both groups were exposed simultaneously to the same batch of *Ornithonyssus bacoti* mites containing infectious *L. sigmodontis* L3 larvae.

### Parasite recovery

Mice were euthanized via an overdose of isofluorane (Abbvie, Wiesbaden, Germany) 35, 60, and 100 days post infection (dpi). To determine adult worm burden, the thoracic cavity of individual mice was flushed with 1 ml of RPMI 1640 medium (PAA Laboratories, Pasching, Austria) which then contained adult worms. Remaining adult worms in the thoracic cavity and the peritoneum were isolated with a dissection probe. After manual separation of worm clusters, the worms were sorted by sex, counted and their lengths determined.

Microfilaria counts were conducted from animals starting at 58 days post infection in weekly intervals up to day 90 pi. For microfilaria counts 50 μl of peripheral blood were taken from the facial vein, added to 1 ml of RBC lyses buffer (eBioscience, San Diego, USA) and incubated for at least 10 min at room temperature, pelleted, and then counted with the aid of a microscope.

### Embryogenesis

To determine the embryonic stages from the uterus, two adult female worms from each mouse were individually transferred into PBS, cut into small pieces and homogenized using a plastic pestle. The embryonic stages were stained using 20 μl Hinkelmann's solution and then 10 μl of this preparation was analyzed under the microscope. Embryonic stages were differentiated as oocytes, pretzel and stretched microfilariae stage as previously described [18].

### Microfilaria injection

Blood from infected cotton rats (*Sigmodon hispidus*) was collected via the facial vein and microfilariae were purified using a sucrose/Percoll density gradient as previously described [19]. In brief, isoosmotic Percoll (Sigma, St. Louis, MO, USA) was prepared by diluting 2.5 M D-sucrose (Sigma) with Percoll 1∶10. Further dilutions of Percoll were made with 0.25 M sucrose to obtain 30% and 25% Percoll. 30% Percoll was laid over a 25% Percoll upon which peripheral blood was carefully added. After centrifugation at 400 g for 35 min the microfilarial layer was collected. Following washing in RPMI, microfilariae were counted and 50,000 microfilariae were inoculated intravenously into the tail vein of naïve ST2-ko mice and corresponding WT controls.

### Splenectomy

Mice were splenectomized under anaesthesia with Ketanest (100 mg/kg) and Rompun (16 mg/kg); a separate group underwent sham surgery to serve as controls. Briefly, the surgical area of the anesthetized mice was sterilized, a small incision was made in the left subcostal area and the peritoneum was opened to exteriorize the spleen. The spleen was removed intact after ligating the splenic bundle at the hilum. For sham surgery, splenic bundle and spleen were left intact. Mice were given three weeks to recover from surgery before they were injected with microfilariae as described in the previous section.

### Isolation of thoracic cavity and spleen cells

Thoracic cavity cells were obtained following lavage with RPMI 1640 media (PAA Laboratories). The first ml of the lavage was collected, worms removed, and cells separated by centrifugation and the supernatant stored at −20°C for cytokine measurements at a later time point. The cells were combined with the cells of a following lavage with 4 ml of RPMI 1640.

Spleen cells were also prepared 35, 60, and 100 days post *L. sigmodontis* infection of WT and ST2-ko mice as previously described [20]. In brief, single cell suspensions were obtained and red blood lysis performed (RBC lyses buffer, eBioscience).

Thoracic cavity and spleen cells were plated at 2×10^6^ cells/ml in enriched media (RPMI 1640 including 10% fetal calf serum, 1% L-glutamine, 100 U/ml penicillin and 100 μg/ml streptomycin (all purchased from PAA Laboratories). Cultures were then left unstimulated or activated with 50 μg/ml crude *L. sigmodontis* antigen (LsAg) or 2.5 ng/ml ConA (Sigma) and cultured at 37°C, 5% CO_2_ for 72 h. Thereafter, cell culture supernatants were removed and stored at −20°C.

### Preparation of *L. sigmodontis* antigen

Preparation of *L. sigmodontis* antigen (LsAg) was performed as previously described [18]. Briefly, freshly isolated adult worms were rinsed in sterile PBS before being mechanically homogenized under sterile conditions. Insoluble material was removed by centrifugation at 300 g for 10 min at 4°C. Protein concentrations of crude extracts were determined using the Advanced Protein Assay (Cytoskeleton, Denver, USA).

### Measurement of cytokines and antibodies by ELISA

Cytokine concentrations were determined within the first ml of thoracic cavity lavage as well as cell culture supernatant by ELISA. IL-4, IL-5 (BD Biosciences, Heidelberg, Germany), IL-10, IL-13, IL-25, IL-33, and IFNγ (all eBioscience) were all measured according to kit protocols. Baseline cytokine production was subtracted from in vitro restimulated samples.

To determine parasite-specific immunoglobulin levels in plasma, plates were coated with 10 μg/ml LsAg overnight. After blocking with PBS/1% BSA, plasma was added in serial dilutions. Following incubation, plates were washed and secondary biotinylated antibodies against IgE, IgG1, IgG2a/2b or IgM (all BD Biosciences) were added. After washing and incubation with Streptavidin-HRP, plates were washed, TMB substrate added and the enzymatic reaction stopped with sulfuric acid. Optical density was measured at 450 nm (Spectramax 240pc Molecular Devices, Biberach, Germany). To measure total IgE, plates were coated with anti-mouse IgE and purified IgE (both BD Biosciences) was used as standard and the assay followed the same protocol as that measuring parasite-specific antibodies.

### Flow cytometric analyses of thoracic cavity and spleen cells

Thoracic cavity and spleen cells were analyzed by flow cytometry. Cells were fixed either by a 20 min incubation with 4% paraformaldehyde or overnight incubation in fixation/permeabilization buffer (eBioscience). Cells were then blocked with PBS/1% BSA including 0.1% Fc block (Sigma). Flow cytometric analysis was performed using a combination of the following conjugates: CD4 PerCP Cy5.5; F4/80 APC; Siglec F PE; B220 APC; anti-IgE FITC; CD335 FITC; DX5 APC, and NIMP FITC (purified from hybridoma supernatants [21] and labelled using the Invitrogen AF488 labelling kit (Invitrogen, Darmstadt, Germany)). CD4^+^ T-cells were identified as CD4^+^, Siglec F low; B-cells as B220^+^, CD4 negative; neutrophils as NIMP high, F4/80 low; eosinophils as Siglec F high, F4/80 low; and NK cells as CD335 high, DX5 high. Macrophage populations were identified as F4/80 high, Siglec F low and alternatively activated macrophages as F4/80 high, Siglec F low, RELMα high. Intracellular staining for RELMα was performed using a two-step staining protocol with rabbit anti-mouse RELMα (Peprotech, Rocky Hill, NJ, USA) followed by a goat anti-rabbit Alexa Fluor 488 conjugated antibody (Invitrogen).

For intracellular cytokine staining isolated thoracic cavity cells were stimulated for 4 hours with either 25 μg/ml LsAg or 50 ng/ml PMA and 1 μg/ml Ionomycin in the presence of Golgi Plug and Golgi Stop (both BD Biosciences). Cells were then incubated overnight in fixation/permeabilization buffer, blocked with PBS/1% BSA including 0.1% Fc Block. Cells were stained with CD4 PerCP Cy5.5 and IL-4 APC, IL-5 PE or IL-10 PE (all eBioscience). In a second staining set, cells were stained with CD335 FITC, DX5 APC, IFNγ PE (all eBioscience).

Flow cytometry was performed using a BD FACS Canto system and subsequently analyzed with FACSDiva 5.1 software (BD Biosciences). During analysis, cut-offs were set using the fluorescence minus one approach.

### Statistics

Statistical analyses were performed with GraphPad Prism software Version 5.03 (GraphPad Software, San Diego, CA, USA). Differences between two unpaired groups were tested for significance with the Mann-Whitney-U-test. P-values of <0.05 were considered statistically significant. Data from different time points are shown as line graphs to improve the clarity, although the data from different time points are not paired.

## Results

### Lack of the ST2 receptor results in a pronounced microfilaremia and in increased adult worm length

In order to investigate the impact of the ST2 receptor on the development of *L. sigmodontis* infection we analyzed the development of patency and microfilarial burden, and quantified adult worm numbers and lengths after: the molt into adult worms (35 dpi), the onset of microfilaremia (60 dpi) and during chronic infection (100 dpi).

**Figure 1:**
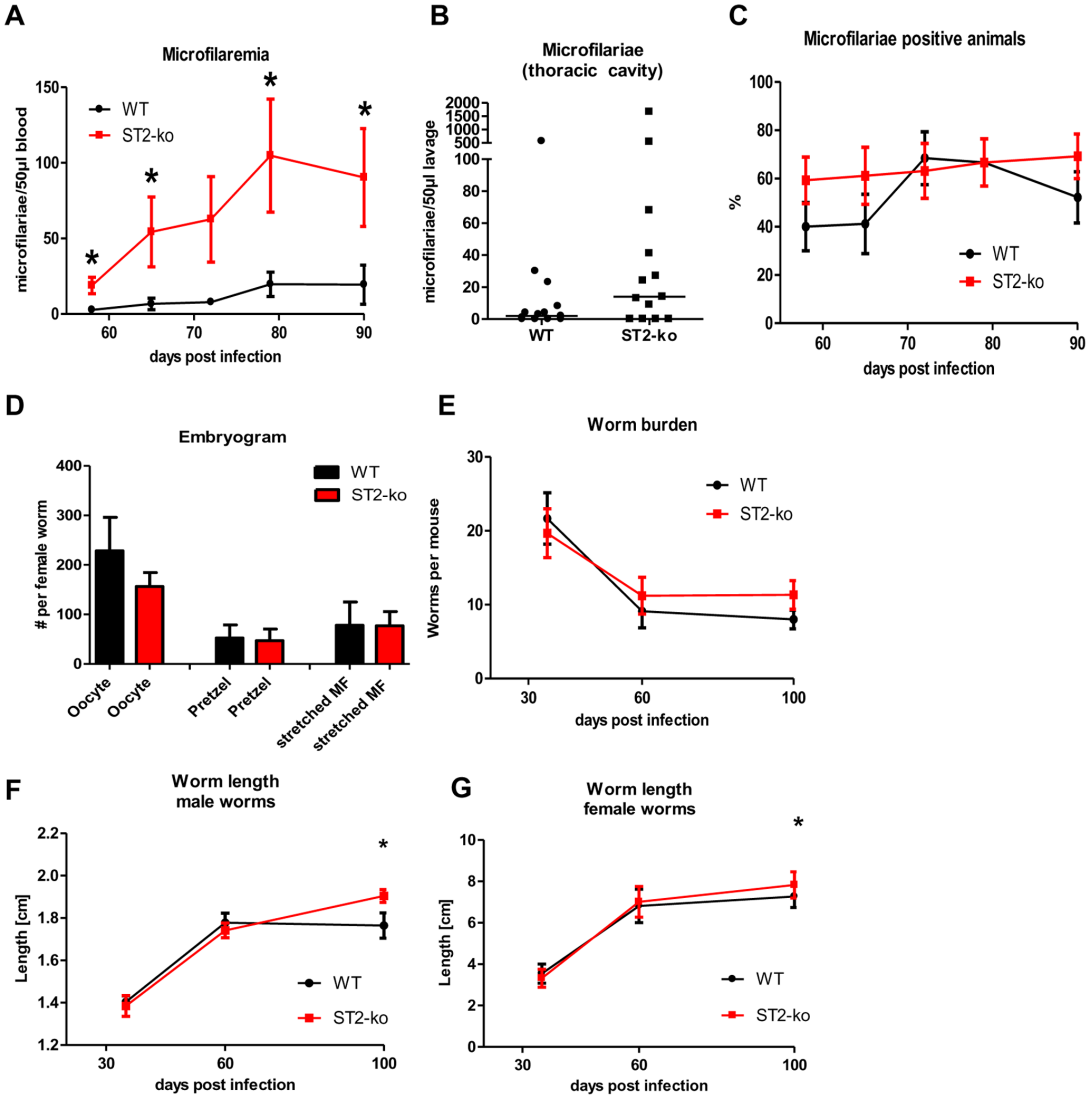
Deficiency in ST2 leads to pronounced microfilaremia and increased adult worm length. A, microfilariae count per 50 μl of peripheral blood of ST2-ko mice and wild type (WT) controls throughout *L. sigmodontis* infection. B, microfilarial burden in the thoracic cavity 60 days post *L. sigmodontis* infection. C, percentage of ST2-ko mice and WT controls that develop patent infections. D, embryogram of female worms 60dpi (6 mice per group and two female worms per mouse). E, adult worm burden in ST2-ko mice and WT controls 35, 60 and 100 dpi, (F and G) length of male and female *L. sigmodontis* worms in WT and ST2-ko mice during infection. A and C show pooled data from three independent experiments with a minimum of 8 mice per group. B shows pooled data from two independent experiments and E-G show representative data of two independent experiments with a minimum of 6 mice per group. Differences were tested for statistical significance by Mann-Whitney-U-test, *p<0.05.

Compared with WT controls, ST2-ko mice had increased numbers of peripheral microfilariae throughout patent infection (Fig. 1A). At the onset of microfilaremia at 58 dpi, average microfilarial numbers in ST2-ko mice were significantly increased compared to WT animals (p = 0.02). The peak of microfilaremia was observed at 79 dpi with significantly increased microfilarial counts in ST2-ko (p = 0.03). At 100 dpi two thirds of the infected BALB/c mice had successfully eliminated microfilariae, whereas two thirds of the ST2-ko mice still had detectable microfilaremia (data not shown). Differences in microfilaremia were not only detected in blood but also in the thoracic cavity since, when compared to WT controls, ST2-ko mice had elevated numbers of microfilariae 60 dpi (Fig. 1B, p = 0.06).

However, the frequency of animals that developed microfilaremia until 90 dpi did not differ between ST2 deficient and WT mice (Fig. 1C). Analysis of embryonic stages within the female worms 60 dpi from ST2-ko and WT mice did not reveal any differences in the number of oocytes, pretzel-stages nor stretched microfilariae (Fig. 1D).

Interestingly, although microfilarial levels were enhanced in ST2-ko mice this did not correspond to elevated adult worm burden (Fig. 1E). This was independent of the time point of investigation as the number of adult worms was equal between both groups directly after the molt to adult worms (35 dpi), at the onset of microfilaremia (60 dpi) and at a late time point of chronic infection (100 dpi). The ratio of female to male worms was not altered by the lack of the ST2 receptor either (data not shown).

As expected, in both groups, worm length increased during the course of infection, with the growth of female *L. sigmodontis* worms being more pronounced than male worms (Fig. 1F, G). Lack of the ST2 receptor did not impact the body length of male and female *L. sigmodontis* adult worms at 35 and 60 dpi. However, at 100 dpi female and male worms were significantly longer in ST2-ko mice compared to worms from WT controls.

### Local cytokine production in acute but not chronically *L. sigmodontis* infected ST2-ko mice is reduced

**Figure 2:**
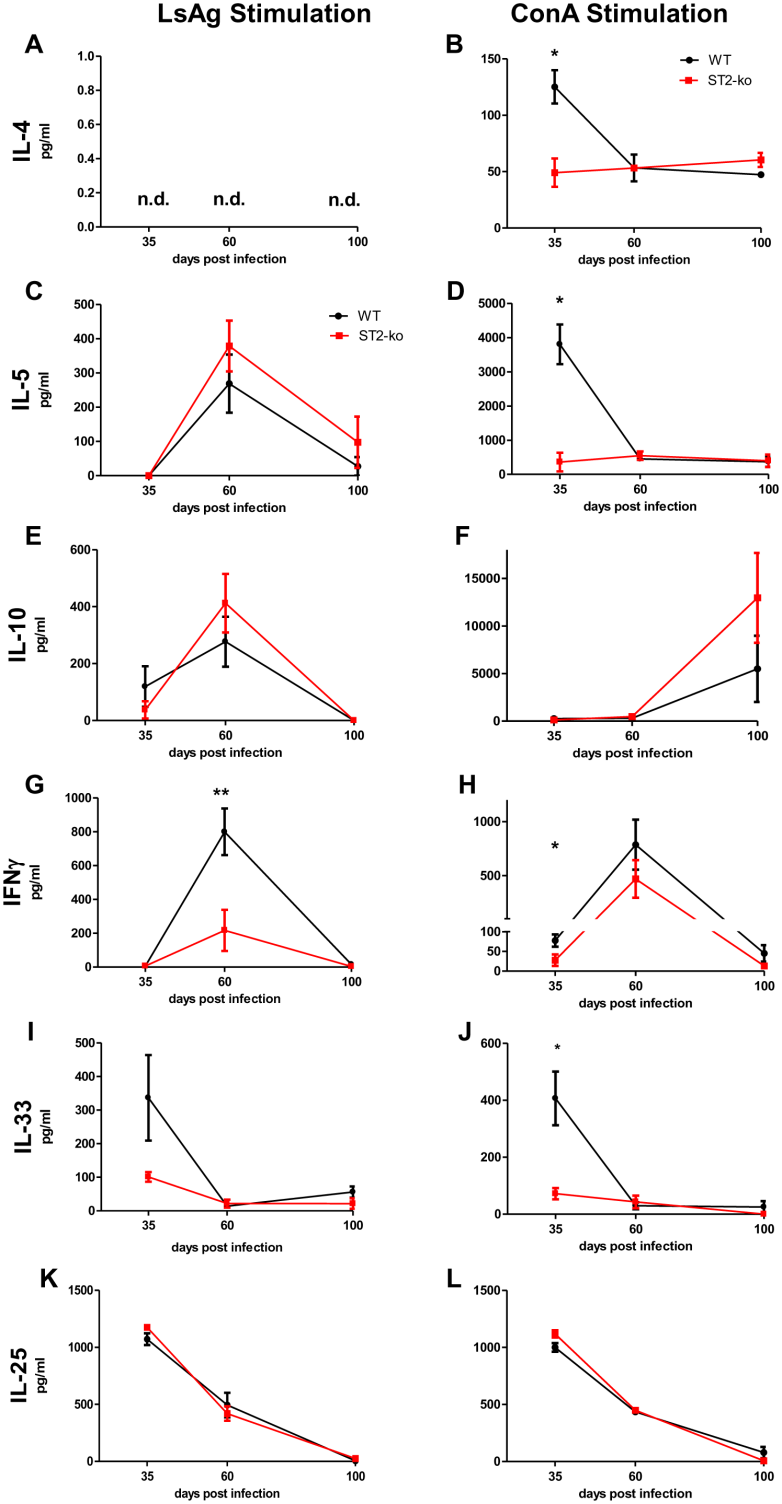
Reduced Th2 cytokine production after in vitro restimulation of thoracic cavity cells of acute, but not chronically *L. sigmodontis* infected ST2-ko mice. Isolated cells from the thoracic cavity of individual *L. sigmodontis* infected wild type (WT) or ST2-ko mice were cultured in vitro with either *L. sigmodontis* antigen (LsAg, left panel) or ConA (right panel). IL-4 (A,B), IL-5 (C,D), IL-10 (E,F), IFNγ (G,H), IL-33 (I,J) and IL-25 (K,L) within the culture supernatants were measured on days 35, 60 or 100 post infection. Data is representative for two independent experiments with at least 5 mice per group. Differences were tested for statistical significance by Mann-Whitney-U-test, *p<0.05, **p<0.01.

As shown above, adult *L. sigmodontis* worms reside within the thoracic cavity. In order to investigate changes in local immune responses during infection, we analyzed the cytokine production of restimulated thoracic cavity cells from *L. sigmodontis* infected ST2 deficient and WT mice. We assessed a panel of cytokines following filarial specific or ConA activation including the Th2-related cytokines IL-4 and IL-5 (Fig. 1A–D), the regulatory cytokine IL-10 (Fig. 2E and F), Th1-related IFNγ (Fig. 1G and H) and Type 2 associated factors IL-33 and IL-25 (Fig. 1I–L).

Interestingly, ConA-induced IL-4 (Fig. 2B), IL-5 (Fig. 2D), IFNγ (Fig. 2H), and IL-33 (Fig. 2J) cytokine release from 35 dpi thoracic cavity cells of ST2 deficient mice were significantly reduced when compared to WT mice. These differences were not observed after the onset of microfilaremia at 60 dpi nor at 100 dpi (Fig. 2B, D, H, J). Moreover, whereas filarial-specific IFN-γ levels were reduced 60 dpi in ST2 deficient mice (Fig. 2G), no differences could be observed in either IL-5 or IL-10 production (Fig. 2C, 2E).

ConA and LsAg induced IL-25 release from thoracic cavity cells of ST2-ko and WT animals was comparable and peaked at 35 dpi and declined with the duration of infection (Fig. 2 K and L). Thus, Type 2 cytokine production was only impaired in cells from infected ST2-ko mice before the onset of microfilaremia and correlated with highest IL-33 concentrations of WT cells during that time point.

**Figure 3:**
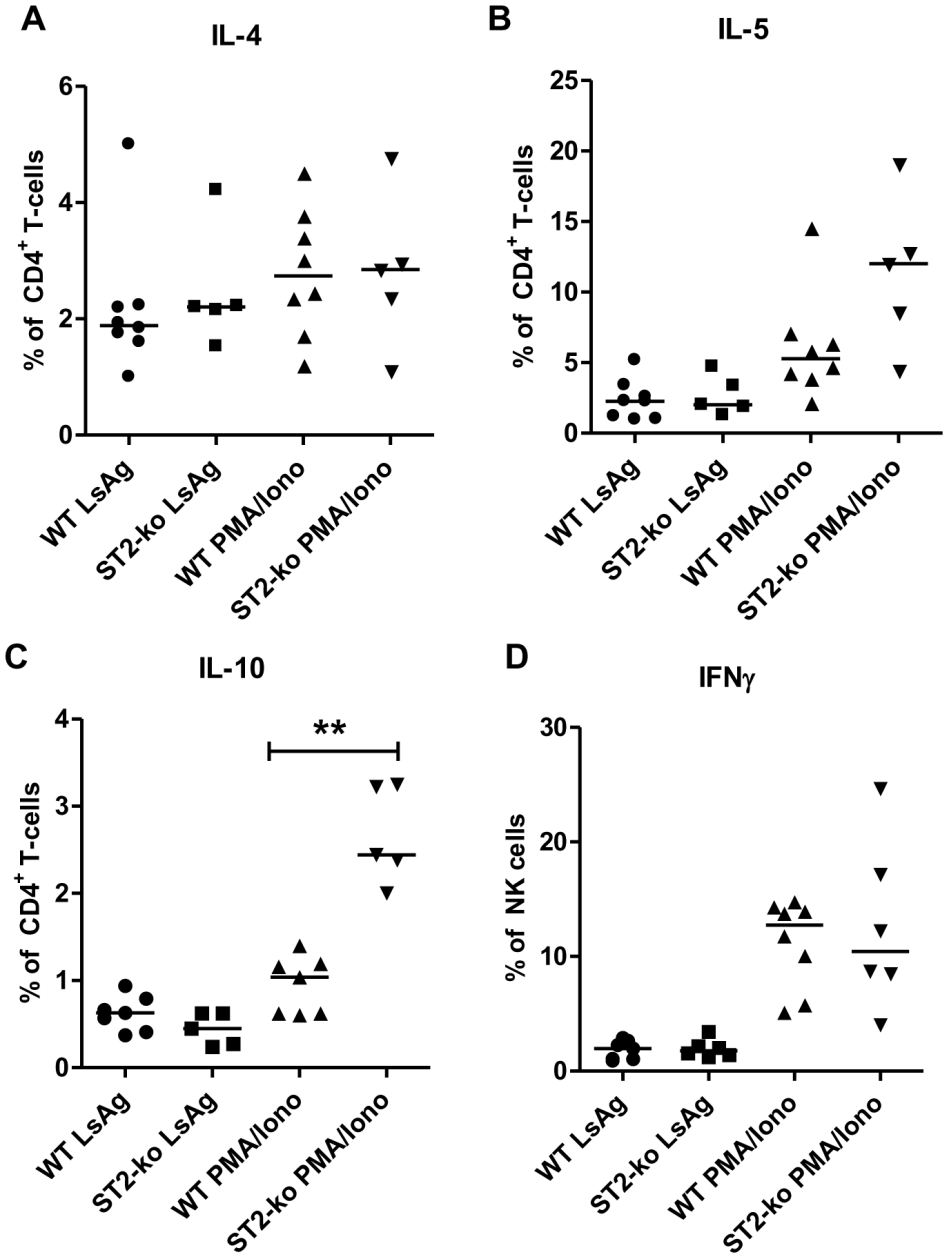
Absence of the ST2 receptor does not impair the frequencies of IL-4, IL-5, IL-10 producing CD4^+^ T-cells and IFNγ^+^ NK cells during *L. sigmodontis* infection. Frequencies of IL-4 (A), IL-5 (B), and IL-10 (C) positive CD4^+^ T- cells as well as IFNγ^+^ NK cells (D) within the thoracic cavity cell populations of 60 days *L. sigmodontis* infected wild type (WT) and ST2-ko mice after 4 h restimulation with LsAg or PMA/Ionomycin. Differences were tested for statistical significance by Mann-Whitney-U-test, **p<0.01.

Intracellular cytokine staining further revealed that LsAg and PMA/Ionomycin induced IL-4, IL-5 and IL-10 production by CD4^+^ T-cells was not impaired in ST2-ko mice during the onset of microfilaremia at 60 dpi (Fig. 3A–C). Indeed, frequencies of IL-10^+^CD4^+^ T-cells (p<0.01) were even higher in ST2-ko mice after PMA/Ionomycin restimulation compared to WT controls. Frequencies of NK cells that were positive for IFNγ did not differ between ST2-deficient and WT animals, independent of their stimulation (Fig. 3D).

### Release of Th2 cytokines at the site of infection is not reduced in *L. sigmodontis* infected ST2-ko mice

Next, we investigated whether there were changes in the cytokine milieu at the site of infection using the thoracic cavity lavage and assessed the levels of several cytokines: IL-4, IL-5, IL-13, IL-25, IL-33, and IFNγ.

**Figure 4:**
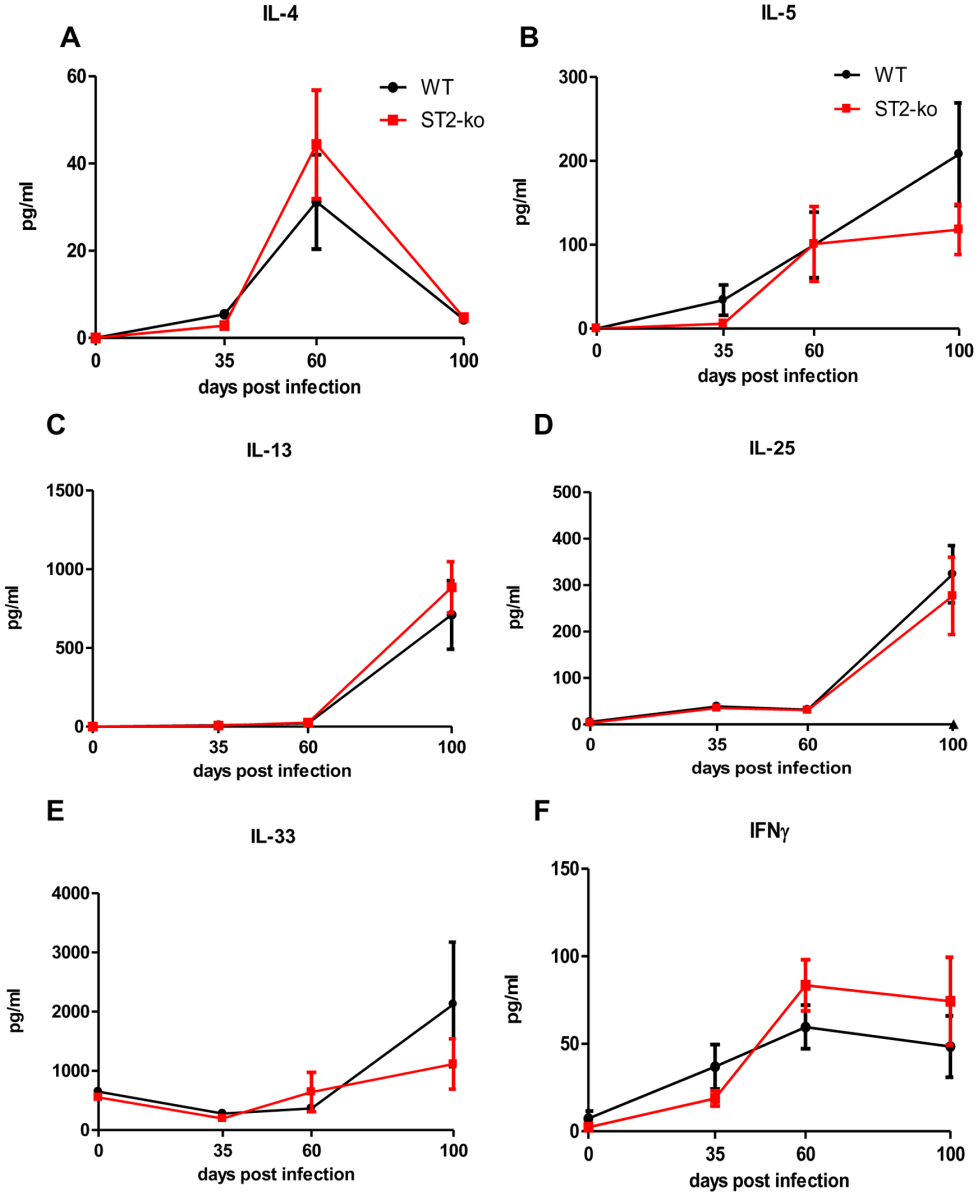
Absence of the ST2 receptor does not change thoracic cavity IL-4, IL-5, IL-13, IL-25, IL-33 and IFNγ levels during *L. sigmodontis* infection. Local concentrations of IL-4 (A), IL-5 (B), IL-13 (C), IL-25 (D), IL-33 (E) and IFNγ (F) in the thoracic cavity lavage prior to infection (day 0) and 35, 60, and 100 days post *L. sigmodontis* infection of wild type (WT) and ST2-ko mice. Data is representative for two independent experiments with at least 5 mice per group. Differences were tested for statistical significance by Mann-Whitney-U-test.

Lack of the ST2 receptor did not lead to an altered production and release of the aforementioned cytokines at 35, 60, and 100 dpi in ST2-ko mice compared to WT controls (Figs. 4A–F). IL-4 cytokine levels (Fig. 4A) peaked with the onset of microfilaremia at 60 dpi, whereas IL-5, IL-13 and IL-25 levels increased with the duration of infection (Figs. 4B–D). Interestingly, IL-33 levels did not increase in comparison to uninfected mice until the late chronic phase of *L. sigmodontis* infection (100 dpi, Fig. 4E). IL-33 production was comparable at all time points between ST2-ko mice and WT controls.

Finally, with regards to thoracic cavity IFNγ levels, these increased with the release of microfilariae and remained elevated until 100 dpi (Fig. 4F). All cytokine levels with the exception of IL-33 in thoracic cavity lavages of naïve animals remained below the detection limit.

### Absence of the ST2 receptor does not impair splenic Th2 cytokine production and systemic antibody levels during *L. sigmodontis* infection

To evaluate differences in the development of systemic Type 2 immune responses following *L. sigmodontis* infection, cell culture supernatants of LsAg and ConA stimulated splenocytes from ST2-ko mice and WT controls were measured via ELISA for Th2 and Th1-related cytokines. Splenocytes from ST2 deficient mice had no impaired production of IL-4, IL-5, IL-10 or IFNγ following ConA stimulation and this was throughout infection (Fig. S1, right panel). In both groups, filarial-specific cytokine release of the aforementioned cytokines was below the detection limit in cultures from mice infected for 35 days and remained low during the onset of microfilaremia at 60 dpi (Fig. S1, left panel). Increased release of the measured cytokines of splenocytes from ST2-ko mice during the late phase of chronic infection (100 dpi) did not reach statistical significance compared to LsAg stimulated spleen cells of WT controls (Fig. S1).

In order to measure the impact of ST2 on the development of systemic antibody levels, production of total IgE as well as the production of LsAg-specific IgE, IgG1, IgG2a/b and IgM was measured in ST2-ko mice and WT controls 35, 60 and 100 dpi. Both the development of total IgE antibody levels (Fig. S2) and filarial-specific IgE, IgG1, IgG2a/b and IgM antibody levels were comparable in WT and ST2 deficient mice at all measured time points (Fig. S3).

### *L. sigmodontis* infected ST2 deficient mice have lower spleen and thoracic cavity cell numbers throughout the infection

**Figure 5:**
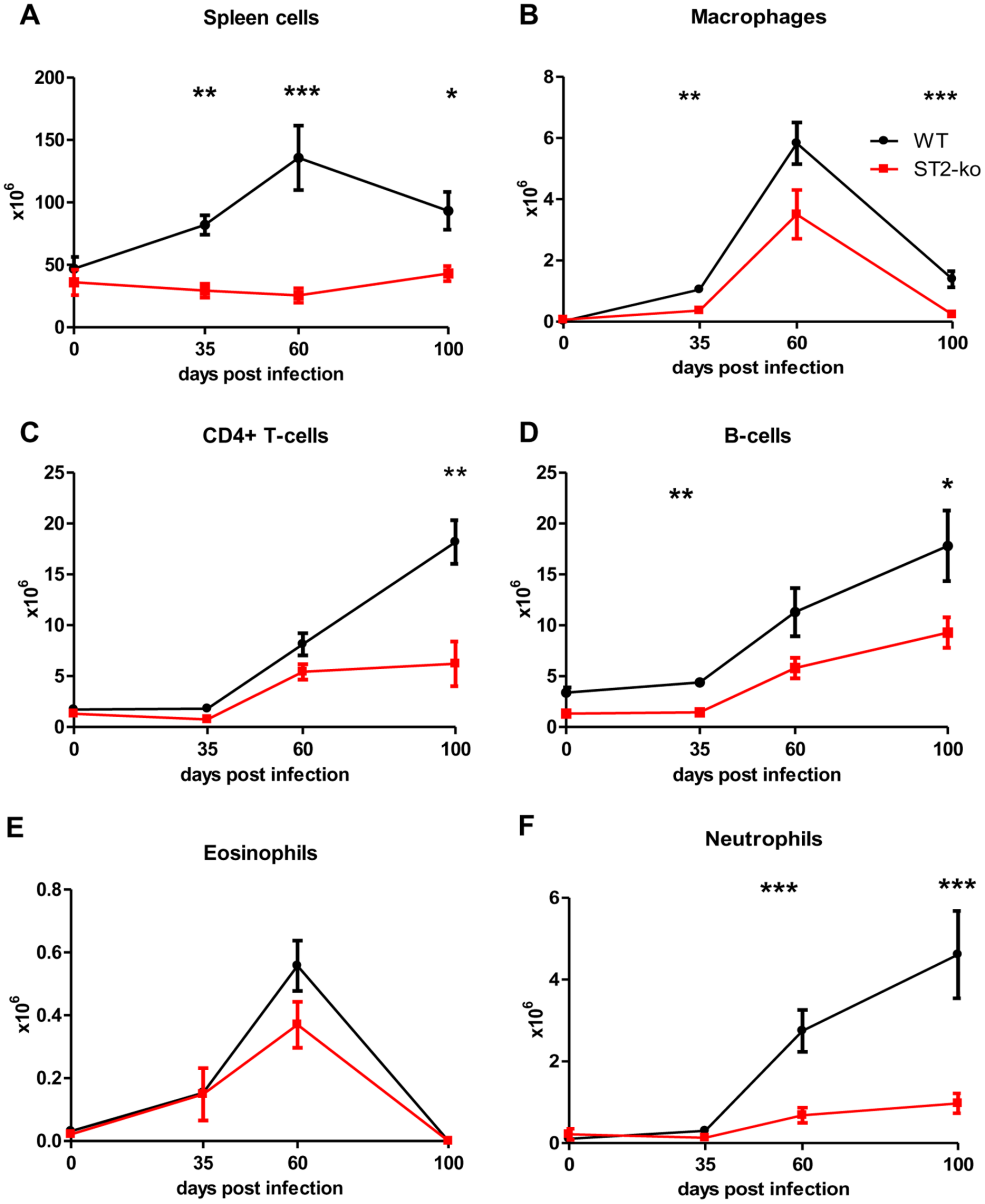
Reduced spleen cell numbers in ST2-ko mice throughout *L. sigmodontis* infection. Different cell populations within the spleen were assessed by flow cytometry in *L. sigmodontis* infected wild type (WT) and ST2-ko mice prior to infection (day 0) and 35, 60 and 100 days post *L. sigmodontis* infection. Absolute number of splenocytes (A), macrophages (B), CD4^+^ T-cells (C), B-cells (D), eosinophils (E) and neutrophils (F) are shown. Data shown is representative for two independent experiments with at least five mice per group. Differences were tested for statistical significance by Mann-Whitney-U-test, *p<0.05, **p<0.01, ***p<0.001.

**Figure 6:**
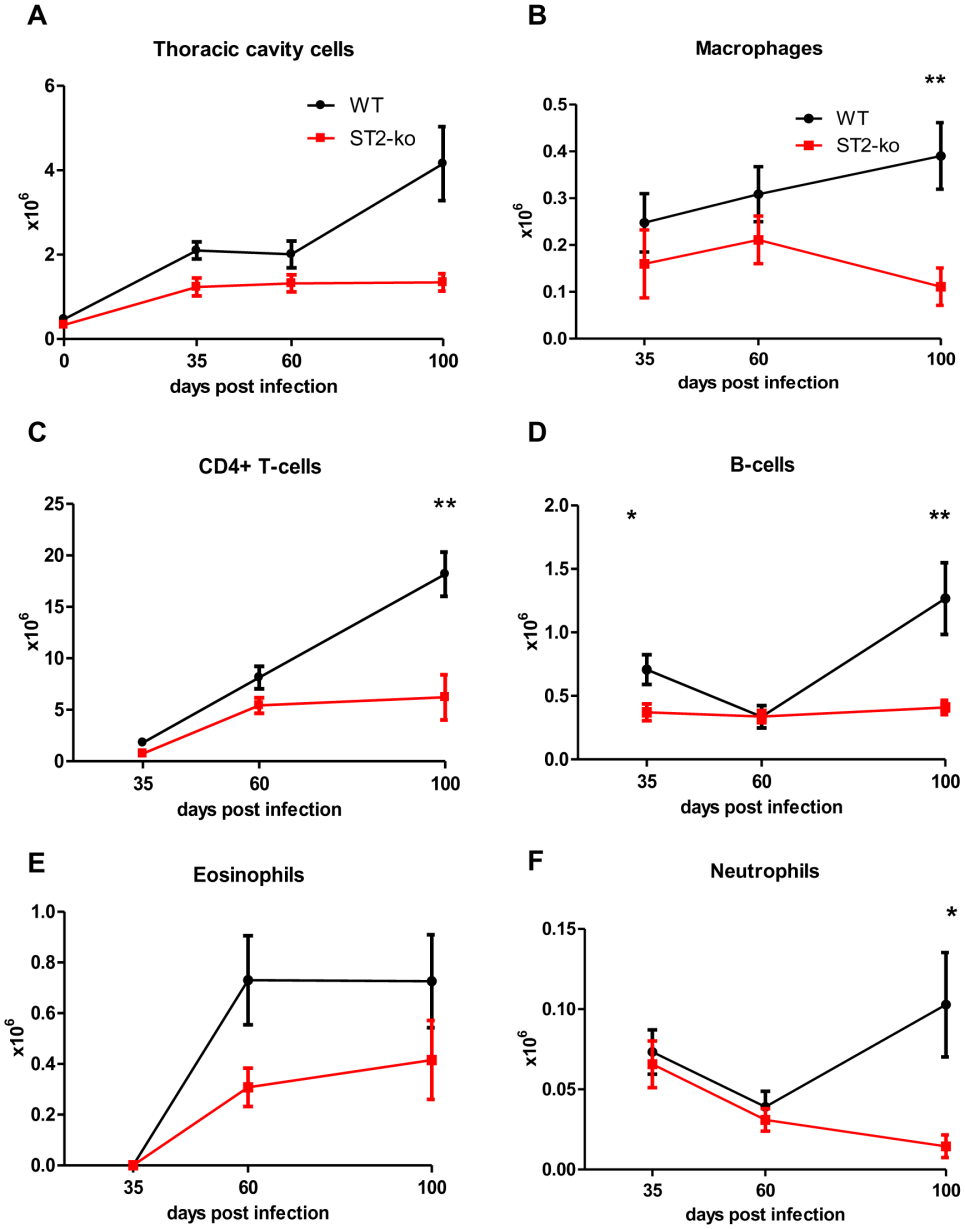
ST2-ko mice have reduced thoracic cavity cell numbers throughout *L. sigmodontis* infection. Cell populations within the thoracic cavity were assessed by flow cytometry in wild type (WT) and ST2-ko mice prior to infection (day 0, only Fig. 6A) and after 35, 60 and 100 days post *L. sigmodontis* infection (Fig. 6B–F). Total number of cells within the thoracic cavity lavage (A), macrophages (B), CD4^+^ T-cells (C), B-cells (D), eosinophils (E) and neutrophils (F) are shown. Data shown is representative for two independent experiments with at least 5 mice per group. Differences were tested for statistical significance by Mann-Whitney-U-test, *p<0.05, **p<0.01.

To examine whether the enhanced microfilariae numbers in ST2-ko mice were associated with different frequencies of cell populations, flow cytometric analyses were performed on spleen and thoracic cavity cells of both groups (Fig. 5 and 6).

No differences in the cellular composition of splenic eosinophils, neutrophils, B-cells, T-cells, and macrophages were observed between WT and ST2 deficient mice that were infected with *L. sigmodontis* for 35, 60 or 100 days (data not shown). However, spleens were significantly smaller in ST2-ko mice compared to WT controls and while the absolute spleen cell number increased in both groups during infection, spleen cell numbers of ST2-ko mice were consistently lower compared to WT controls (Fig. 5A). Accordingly, absolute numbers of macrophages and B-cells were significantly lower in the ST2-ko mice 35 dpi, whereas there was no statistical significant difference regarding CD4^+^ T-cells, eosinophils and neutrophils at that time point (Fig. 5 B–F). 60 dpi, when microfilariae begin to enter the peripheral blood, spleen cell numbers peaked in WT animals and ST2-ko mice had significantly reduced total numbers of neutrophils and tended to have reduced numbers of B- and T-cells, eosinophils and macrophages. 100 dpi the absolute numbers of CD4^+^ T-cells, macrophages, B-cells and neutrophils in the spleens of ST2-ko animals were significantly lower compared to WT controls. Increased numbers of neutrophils were only observed in the spleen after the onset of peripheral microfilaremia. Neutrophils were present in naïve mice at very low levels suggesting that systemic immune responses to the filarial infection mediated the recruitment of neutrophils to the spleen. In the absence of *L. sigmodontis* infection spleens of ST2-ko mice remained significantly smaller compared to WT mice (Fig. 5A). In both groups, the numbers of tested cell types were lower in uninfected mice compared to infected animals (Fig. 5B–F).

Analogous to the spleen cells, we observed lower numbers of thoracic cavity cells in the ST2-ko mice (Fig. 6A), but no differences in the frequencies of CD4^+^ T-cells, macrophages, eosinophils, neutrophils and B-cells (data not shown). Absolute cell numbers of CD4^+^ T-cells and eosinophils increased with infection time, while macrophage, neutrophil and B-cell numbers remained at similar levels between 35 and 100 dpi (Fig. 6B–F). 35 dpi cell numbers of macrophages (p>0.05) and B-cells (p<0.05) were higher in WT controls compared to ST2 deficient mice. Absolute neutrophil, eosinophil, B-cell, and CD4^+^ T-cell numbers did not show any statistical differences between the groups on 60 dpi. Numbers of macrophages (Fig. 6B) were not statistically different at 60 dpi between both groups and did not reveal any differences in their expression levels of RELMα (Fig. S4), suggesting a similar induction of alternatively activated macrophages. 100 dpi all measured cell type numbers were lower in ST2-ko mice, reaching statistical significance for macrophages (p<0.01), T-cells (p<0.01), B-cells (p<0.01), and neutrophils (p = 0.01).

### Spleen-mediated removal of injected microfilariae is impaired in ST2-ko mice

Since the embryogram did not reveal an increased production of microfilariae by female adult worms in ST2 deficient mice (Fig. 1D), we injected microfilariae into naïve WT and ST2-ko mice to investigate whether lack of ST2 impairs the clearance rate of peripheral microfilariae.

**Figure 7:**
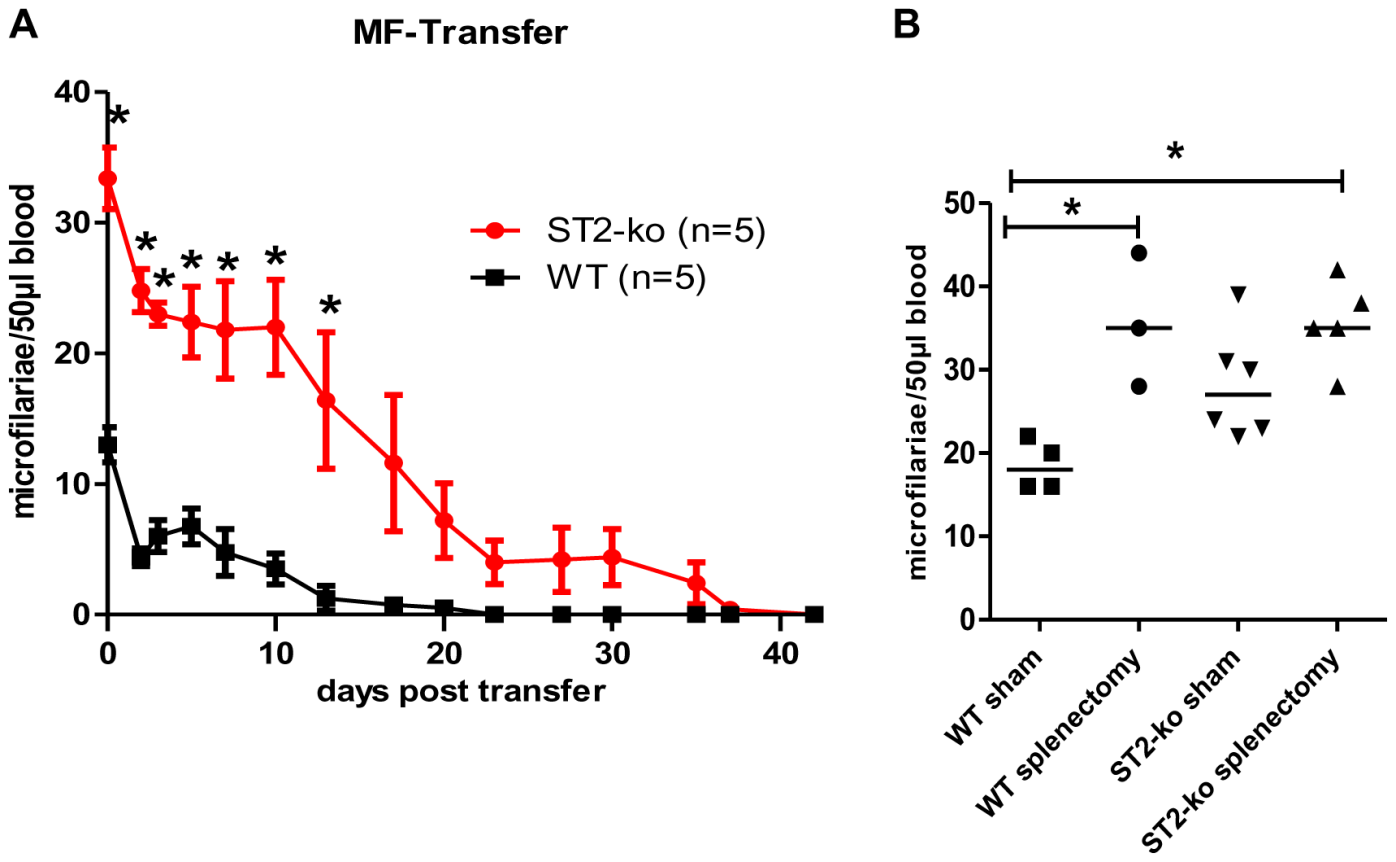
ST2-ko mice have a delayed splenic clearance of transferred microfilariae. Kinetics of blood microfilariae numbers in ST2-ko mice and wild type (WT) controls after i.v. inoculation of 50,000 microfilariae (A). Blood microfilariae counts in splenectomized and sham-treated ST2-ko mice and WT controls one hour after inoculation with 50,000 microfilariae per mouse (B). Differences were tested for statistical significance by Mann-Whitney-U-test, *p<0.05. Representative data shown in (A) is from two independent experiments with at least 6 mice per group.

As shown in Fig. 7A, ST2-ko mice have a delayed clearance of injected microfilariae compared to WT controls. As early as 1 h after microfilariae inoculation, ST2-ko mice retained significantly more microfilariae compared to WT controls (WT: average of 13 microfilariae/50 μl blood, ST2-ko: 33 microfilariae/50 μl blood). Similar results were obtained in a repeat experiment. Significantly increased numbers of microfilariae in the peripheral blood of ST2-ko mice was maintained until 13 days post inoculation. Indeed, whereas all ST2-ko mice were still microfilariae positive 13 days post injection, 50% of the WT controls had no detectable peripheral blood microfilariae at this time point. On day 23 post inoculation no microfilariae were detectable in WT animals but 60% of the ST2-ko mice still had viable circulating microfilariae. On day 42 post inoculation all ST2-ko mice had successfully cleared all peripheral microfilariae. Of note, comparing the time required to clear 50% of the microfilarial load (T1/2) revealed that ST2-ko mice required on average 15 days whereas it took ∼12 days in WT animals (data not shown).

As microfilariae can be found in the spleen after inoculation, we investigated whether the impaired clearance of injected microfilariae in ST2 deficient mice was mediated by the spleen. Thus, naïve mice were splenectomized prior to inoculation with microfilariae. One hour after microfilariae injection, splenectomized WT mice had microfilariae levels that were comparable with splenectomized ST2-ko animals (both groups median 35 microfilariae/50 μl blood) and were significantly higher than the levels seen in WT controls which had undergone sham surgery (median 18 microfilariae/50 μl blood; Fig. 7B). Sham treated ST2 deficient mice had microfilariae levels (median 27 microfilariae/50 μl blood) that were significantly increased compared to sham treated WT controls, but lower than splenectomized groups (Fig. 7B).

## Discussion

Current knowledge surrounding the impact of ST2/IL-33 interactions on helminth infections is based on studies using intestinal helminths [9], [13], [14], [15], [22], [23], [24]. We provide here the first evidence that the ST2 receptor is important for the control of microfilariae during filarial infection. The observed increased microfilarial load in ST2 deficient mice was due to an impaired splenic clearance of microfilariae while filarial embryogenesis was not improved in ST2-ko mice. Accordingly, the lack of ST2 did not alter the frequency of microfilariae positive mice compared to WT controls. While the lack of the ST2 receptor did not affect adult *L. sigmodontis* worm burden and the clearance of adult worms over time, adult worm length was significantly prolonged in ST2-ko mice during chronic infection (100 dpi), suggesting a growth benefit of adult worms in the absence of ST2 signaling.

Similar results have been reported by Scalfone et al. who described an increase in *T. spiralis* larval burden but no differences in adult worm burden in ST2 deficient mice [15]. In correlation, ST2 deficient mice had an equivalent *Nippostrongylus brasiliensis* worm burden and expulsion rate compared to WT controls [9]. Additional studies investigating helminth-induced immune responses in ST2-ko mice did not examine whether ST2 receptor deficiency has an influence on the adult worm burden or fertility [13], [24]. Thus it remains unclear to what extent interactions with ST2 are involved in protective immune responses against various helminth species.

IL-33, the ligand for ST2, is an alarmin, released by necrotic cells after damage or injury, which drives Type 2 immune responses. Interestingly, such damage and consequential release of IL-33 by epithelial cells, alveolar macrophages and inflammatory dendritic cells has been shown when *N. brasiliensis* larvae migrate through the lung [2]. During *L. sigmodontis* infection, however, only low amounts of the Type 2 cytokines IL-5 and IL-13 were measured in the thoracic cavities of WT and ST2-ko mice before and during the onset of microfilaremia (35 and 60 dpi, respectively) a pattern also reflected in the amounts of released IL-33.

Nevertheless, in vitro restimulation of thoracic cavity cells with the mitogen ConA, that activates the majority of T cells, induced dominant IL-4 and IL-5 cytokine production on 35 dpi from WT cells, but these cytokine responses were significantly reduced in ST2-ko mice. The peak in IL-4 and IL-5 production coincided with peak IL-33 concentrations. Interestingly, a potential cellular source of IL-33 and IL-25 are mast cells [4] and since such cells can be activated by ConA [25], they could be responsible for the production of IL-33 and IL-25 in these co-culture experiments. This observation agrees with previous studies demonstrating that IL-33/ST2 interactions amplify Type 2 immune responses [1], [14] and lack of the ST2 receptor reduces Th2 skewing [13]. The disparity in Type 2 cytokine profiles observed between thoracic cavity lavage and in vitro restimulation may be explained by the differences in the intensity of activation and the immunological milieu.

Parasite specific immune responses are diminished during filarial infection [26]. After the molt into adult worms (35 dpi), parasite specific immune responses were low after in vitro restimulation of thoracic cavity cells and cytokine levels in the thoracic cavity lavage were close to the detection limit. Filarial-specific immune responses increased after the onset of patent infections in WT mice and when compared to ST2-ko mice there were no differences in local (cytokine levels within the thoracic cavity lavage, after restimulation of thoracic cavity cells and frequencies of IL-4^+^ and IL-5^+^ CD4^+^ T-cells) and systemic (spleen cell cytokine production after restimulation and parasite-specific and total IgE levels) Type 2 immune responses.

Helminth-induced feedback loops involving IL-25 production and activation of type 2 innate lymphoid cells (ILC2s) that initiate Type 2 immune responses may further compensate for the lack of ST2 signaling. A redundant role for IL-25/IL-33 has been demonstrated in the expulsion of *N. brasiliensis*, as only animals that are deficient for both the ST2 and IL-25 receptors had a delayed worm clearance [9]. During *L. sigmodontis* infection, levels of IL-13 and IL-25 within the thoracic cavity lavage were comparable between ST2 deficient and WT mice and had similar kinetics as IL-33. Thus, similar induction of Type 2 immune responses by ST2 and WT mice during chronic *L. sigmodontis* infection may be due to the redundant roles of IL-25 and IL-33 in ST2-ko mice. Among the ST2 expressing cell types that may accumulate during *L. sigmodontis* infection in the thoracic cavity and induce Type 2 immune responses are Th2 cells, mast cells, eosinophils, basophils, macrophages, and ILC2s, although the latter were not yet described during *L. sigmodontis* infection.

A primary finding in this study was the elevation of microfilariae levels in ST2-ko mice. IL-4 and IL-5 are known to be involved in protective immune responses against filariae and their absence lead to elevated microfilarial burdens in *L. sigmodontis* infected mice [11], [21]. Since we observed a reduction in Type 2 cytokine release at early time points during *L. sigmodontis* infection in ST2-ko mice, such down-regulation may provide an immunological milieu that facilitates microfilariae survival. In correlation, the Th1 cytokine IFNγ is also involved in the protection against *L. sigmodontis* and lack of IFNγ results in an increased microfilarial burden [27], [28]. Interestingly, thoracic cavity cells from *L. sigmodontis* infected ST2-ko mice produced less IFNγ after in vitro restimulation at 35 and 60 dpi. NK cells that can respond to IL-33 with enhanced IFNγ production [29] were not impaired in cells from *L. sigmodontis* infected ST2-ko mice. Based on the similar embryogenesis of female adult worms and comparable cytokine milieu at the site of infection, we concluded that the increased microfilaremia in ST2-ko mice is not primarily caused by differences in the production of Type 2 cytokines or IFNγ.

Similar to the cytokine milieu within the thoracic cavity, the cellular composition in the thoracic cavity was not altered by the loss of the ST2 receptor during *L. sigmodontis* infection. However, total thoracic cavity and spleen cell numbers were consistently reduced in ST2-ko mice. These included lower absolute cell numbers of CD4^+^ T-cells, B-cells, macrophages, neutrophils, and eosinophils in ST2-ko mice and such deficits may facilitate worm development and microfilariae production and survival, especially since both eosinophils and neutrophils are known to mediate protection against filarial infections [12], [21], [27], [28], [30], [31]. While those studies demonstrated that both eosinophils and neutrophils are protective against adult filarial worms, Simons et al. showed that it was eosinophils, but not neutrophils, that impaired microfilariae survival after either microfilariae injection or the implantation of microfilariae releasing *Brugia malayi* female adult worms [32], [33]. Although eosinophil numbers were consistently lower in the spleen and thoracic cavity of *L. sigmodontis*-infected ST2-ko mice after the onset of microfilaremia, those differences did not reach statistical significance, suggesting that functional differences of eosinophils in both strains may promote microfilaremia. Different to the *Brugia malayi* model, previous publications [21], [28] also suggest that neutrophils may be important for protective immune responses against the microfilarial stage during natural *L. sigmodontis* infection. Accordingly, neutrophil numbers increased by ten-fold in spleens of WT mice after the onset of microfilaremia, whereas this increase was less prominent in ST2-ko mice.

Besides eosinophils and neutrophils, the significant reduction of B-cells in ST2-ko mice before the onset of microfilaremia may also account for the observed increased microfilarial load. Mice that lack B1-cells have an increased susceptibility to filarial infections and develop higher *L. sigmodontis* microfilaremia and adult worm burdens. Indeed, IgM produced by B1 cells is thought to improve the immunity against the microfilarial stage [34], [35], [36]. Although ST2-ko mice had lower absolute numbers of B-cells, levels of parasite-specific IgM and total IgM did not differ with *L. sigmodontis* infection in ST2-ko and WT mice nor in naïve mice. Similarly, parasite-specific IgE, IgG1 and IgG2a/b levels were comparable between both groups, suggesting that antibodies were not responsible for the observed increased microfilaremia in ST2-ko mice.

To expand upon our in vivo findings of elevated microfilariae in the absence of ST2, we performed clearance experiments by injecting microfilariae into naïve WT and ST2-ko mice. After only one hour, microfilariae levels in naïve ST2-ko mice were significantly higher compared to WT controls. Following immune responses to the microfilariae seemed comparable in both mouse strains, as there were only minor differences in the T1/2 of microfilariae clearance. As a result of the initial higher microfilarial burden in ST2-ko mice, BALB/c WT mice eliminated injected microfilariae from peripheral blood within three to four weeks, as was previously described [37], whereas 60% of ST2-ko mice had circulating microfilariae in the peripheral blood at that time point. This suggests that ST2-ko mice are more hospitable to injected microfilariae based on a mechanism in which ST2 influences the survival of microfilariae immediately after release. It is generally assumed that the clearance of peripheral microfilariae takes place in the liver, lung and spleen [38], [39], [40], [41]. As we observed a significant reduction in total numbers of splenocytes in ST2-ko mice throughout *L. sigmodontis* infection and in naïve mice, we hypothesized that this may impair the initial protective immune response to microfilariae. Indeed, the involvement of the spleen in the removal of injected microfilariae could be shown in splenectomized mice that harbored higher numbers of microfilariae compared to sham treated WT and ST2 deficient controls. Importantly, after splenectomy, microfilarial loads were equivalent in ST2 deficient and WT mice, suggesting that the spleen mediated the observed differences in the microfilarial load.

Future studies are required to investigate whether the impaired splenic clearance of microfilariae of ST2 deficient mice is due to the anatomic spleen structure that may physically impair the removal of microfilariae from the peripheral blood or whether immediate protective immune responses are impaired. As we did not observe differences in IgM concentrations, it could be speculated that changes in complement factors are involved and impair the splenic removal of microfilariae in ST2-ko mice.

In conclusion, our study investigated for the first time the impact of ST2 on the development of a non-enteric helminth using ST2 deficient mice and infections with the filarial nematode *L. sigmodontis*. The absence of the ST2 receptor had no effect on protective immune responses against the adult stage of *L. sigmodontis*, but led to a significantly higher microfilarial burden. The increased microfilarial burden in ST2-ko mice did not correlate with an impaired Th2 cytokine response after the onset of microfilaremia, but was shown to be due to an impaired splenic clearance of microfilariae.

## Supporting Information

Figure S1 **Lack of ST2 does not impair splenic Th2 cytokine production during** ***L. sigmodontis*** **infection.** Isolated cells from the spleens of individual *L. sigmodontis* infected wild type (WT) and ST2-ko mice were cultured in vitro with either *L. sigmodontis* antigen (LsAg, left panel) or ConA (right panel). IL-4 (A, B), IL-5 (C, D), IL-10 (E, F), and IFNγ (G, H) within the cell culture supernatants were measured before infection (day 0) and on days 35, 60 or 100 post infection. Data is representative for two independent experiments for each measured time point with at least 5 mice per group. Differences were tested for statistical significance by Mann-Whitney-U-test, *p<0.05.
(TIF)

Figure S2 **No differences in the production of total IgE between ST2-ko mice and wild type controls during** ***L. sigmodontis*** **infection.** Total IgE antibody levels in plasma of ST2-ko mice and wild type (WT) controls on 35, 60 and 100 days post *L. sigmodontis* infection as well as naïve animals. Differences were tested for statistical significance by Mann-Whitney-U-test.
(TIF)

Figure S3 **No differences in the production of filarial specific antibodies between ST2-ko mice and wild type controls.** Optical density (OD) of IgG1 (A–C), IgG2a/b (D–F), IgM (G–I) and IgE (J–L) in plasma of ST2-ko mice and wild type (WT) controls 35, 60 and 100 days post *L. sigmodontis* infection. Differences were tested for statistical significance by Mann-Whitney-U-test.
(TIF)

Figure S4 **Similar expression of RELMα on thoracic cavity macrophages of ST2-ko mice and wild type controls.** RELMα mean fluorescence intensity (MFI) from macrophages of naïve and 60 day *L. sigmodontis* infected wild type (WT) and ST2-ko mice.
(TIF)

## References

[1] Schmitz J, Owyang A, Oldham E, Song Y, Murphy E, et al (2005) IL-33, an interleukin-1-like cytokine that signals via the IL-1 receptor-related protein ST2 and induces T helper type 2-associated cytokines. Immunity 23: 479–490. 16286016 10.1016/j.immuni.2005.09.015

[2] Wills-Karp M, Rani R, Dienger K, Lewkowich I, Fox JG, et al (2012) Trefoil factor 2 rapidly induces interleukin 33 to promote type 2 immunity during allergic asthma and hookworm infection. J Exp Med 209: 607–622. 22329990 10.1084/jem.20110079 PMC3302229

[3] Hardman CS, Panova V, McKenzie AN (2013) IL-33 citrine reporter mice reveal the temporal and spatial expression of IL-33 during allergic lung inflammation. Eur J Immunol 43: 488–498. 23169007 10.1002/eji.201242863 PMC3734634

[4] Hsu CL, Neilsen CV, Bryce PJ (2010) IL-33 is produced by mast cells and regulates IgE-dependent inflammation. PLoS ONE 5: e11944. 20689814 10.1371/journal.pone.0011944 PMC2914748

[5] Pichery M, Mirey E, Mercier P, Lefrancais E, Dujardin A, et al (2012) Endogenous IL-33 is highly expressed in mouse epithelial barrier tissues, lymphoid organs, brain, embryos, and inflamed tissues: in situ analysis using a novel Il-33-LacZ gene trap reporter strain. J Immunol 188: 3488–3495. 22371395 10.4049/jimmunol.1101977

[6] Lohning M, Stroehmann A, Coyle AJ, Grogan JL, Lin S, et al (1998) T1/ST2 is preferentially expressed on murine Th2 cells, independent of interleukin 4, interleukin 5, and interleukin 10, and important for Th2 effector function. Proc Natl Acad Sci U S A 95: 6930–6935. 9618516 10.1073/pnas.95.12.6930 PMC22690

[7] Moritz DR, Rodewald HR, Gheyselinck J, Klemenz R (1998) The IL-1 receptor-related T1 antigen is expressed on immature and mature mast cells and on fetal blood mast cell progenitors. J Immunol 161: 4866–4874. 9794420

[8] Suzukawa M, Iikura M, Koketsu R, Nagase H, Tamura C, et al (2008) An IL-1 cytokine member, IL-33, induces human basophil activation via its ST2 receptor. J Immunol 181: 5981–5989. 18941187 10.4049/jimmunol.181.9.5981

[9] Neill DR, Wong SH, Bellosi A, Flynn RJ, Daly M, et al (2010) Nuocytes represent a new innate effector leukocyte that mediates type-2 immunity. Nature 464: 1367–1370. 20200518 10.1038/nature08900 PMC2862165

[10] Kurowska-Stolarska M, Stolarski B, Kewin P, Murphy G, Corrigan CJ, et al (2009) IL-33 amplifies the polarization of alternatively activated macrophages that contribute to airway inflammation. J Immunol 183: 6469–6477. 19841166 10.4049/jimmunol.0901575

[11] Volkmann L, Bain O, Saeftel M, Specht S, Fischer K, et al (2003) Murine filariasis: interleukin 4 and interleukin 5 lead to containment of different worm developmental stages. Med Microbiol Immunol (Berl) 192: 23–31. 12592560 10.1007/s00430-002-0155-9

[12] Specht S, Saeftel M, Arndt M, Endl E, Dubben B, et al (2006) Lack of eosinophil peroxidase or major basic protein impairs defense against murine filarial infection. Infect Immun 74: 5236–5243. 16926417 10.1128/IAI.00329-06 PMC1594830

[13] Townsend MJ, Fallon PG, Matthews DJ, Jolin HE, McKenzie AN (2000) T1/ST2-deficient mice demonstrate the importance of T1/ST2 in developing primary T helper cell type 2 responses. J Exp Med 191: 1069–1076. 10727469 10.1084/jem.191.6.1069 PMC2193113

[14] Humphreys NE, Xu D, Hepworth MR, Liew FY, Grencis RK (2008) IL-33, a potent inducer of adaptive immunity to intestinal nematodes. J Immunol 180: 2443–2449. 18250453 10.4049/jimmunol.180.4.2443

[15] Scalfone LK, Nel HJ, Gagliardo LF, Cameron JL, Al-Shokri S, et al (2013) Participation of MyD88 and interleukin-33 as innate drivers of Th2 immunity to Trichinella spiralis. Infect Immun 81: 1354–1363. 23403558 10.1128/IAI.01307-12 PMC3639596

[16] Allen JE, Adjei O, Bain O, Hoerauf A, Hoffmann WH, et al (2008) Of mice, cattle, and humans: the immunology and treatment of river blindness. PLoS Negl Trop Dis 2: e217. 18446236 10.1371/journal.pntd.0000217 PMC2323618

[17] Hoffmann W, Petit G, Schulz-Key H, Taylor D, Bain O, et al (2000) Litomosoides sigmodontis in mice: reappraisal of an old model for filarial research. Parasitol Today 16: 387–389. 10951598 10.1016/s0169-4758(00)01738-5

[18] Ziewer S, Hübner MP, Dubben B, Hoffmann WH, Bain O, et al (2012) Immunization with L. sigmodontis Microfilariae Reduces Peripheral Microfilaraemia after Challenge Infection by Inhibition of Filarial Embryogenesis. PLoS Negl Trop Dis 6: e1558. 22413031 10.1371/journal.pntd.0001558 PMC3295809

[19] Chandrashekar R, Rao UR, Rajasekariah GR, Subrahmanyam D (1984) Separation of viable microfilariae free of blood cells on Percoll gradients. J Helminthol 58: 69–70. 6538892 10.1017/s0022149x00028078

[20] Hübner MP, Stocker JT, Mitre E (2009) Inhibition of type 1 diabetes in filaria-infected non-obese diabetic mice is associated with a T helper type 2 shift and induction of FoxP3+ regulatory T cells. Immunology 127: 512–522. 19016910 10.1111/j.1365-2567.2008.02958.x PMC2729528

[21] Al-Qaoud KM, Pearlman E, Hartung T, Klukowski J, Fleischer B, et al (2000) A new mechanism for IL-5-dependent helminth control: neutrophil accumulation and neutrophil-mediated worm encapsulation in murine filariasis are abolished in the absence of IL-5. Int Immunol 12: 899–908. 10837417 10.1093/intimm/12.6.899

[22] Hung LY, Lewkowich IP, Dawson LA, Downey J, Yang Y, et al.. (2012) IL-33 drives biphasic IL-13 production for noncanonical Type 2 immunity against hookworms. Proc Natl Acad Sci U S A. 10.1073/pnas.1206587110 PMC3538196 23248269

[23] Yasuda K, Muto T, Kawagoe T, Matsumoto M, Sasaki Y, et al (2012) Contribution of IL-33-activated type II innate lymphoid cells to pulmonary eosinophilia in intestinal nematode-infected mice. Proc Natl Acad Sci U S A 109: 3451–3456. 22331917 10.1073/pnas.1201042109 PMC3295287

[24] Hoshino K, Kashiwamura S, Kuribayashi K, Kodama T, Tsujimura T, et al (1999) The absence of interleukin 1 receptor-related T1/ST2 does not affect T helper cell type 2 development and its effector function. J Exp Med 190: 1541–1548. 10562328 10.1084/jem.190.10.1541 PMC2195706

[25] Hook WA, Dougherty SF, Oppenheim JJ (1974) Release of histamine from hamster mast cells by concanavalin A and phytohemagglutinin. Infect Immun 9: 903–908. 4132912 10.1128/iai.9.5.903-908.1974 PMC414904

[26] Maizels RM, Balic A, Gomez-Escobar N, Nair M, Taylor MD, et al (2004) Helminth parasites—masters of regulation. Immunol Rev 201: 89–116. 15361235 10.1111/j.0105-2896.2004.00191.x

[27] Saeftel M, Arndt M, Specht S, Volkmann L, Hoerauf A (2003) Synergism of gamma interferon and interleukin-5 in the control of murine filariasis. Infect Immun 71: 6978–6985. 14638787 10.1128/IAI.71.12.6978-6985.2003 PMC308906

[28] Saeftel M, Volkmann L, Korten S, Brattig N, Al-Qaoud K, et al (2001) Lack of interferon-gamma confers impaired neutrophil granulocyte function and imparts prolonged survival of adult filarial worms in murine filariasis. Microbes Infect 3: 203–213. 11358714 10.1016/s1286-4579(01)01372-7

[29] Bourgeois E, Van LP, Samson M, Diem S, Barra A, et al (2009) The pro-Th2 cytokine IL-33 directly interacts with invariant NKT and NK cells to induce IFN-gamma production. Eur J Immunol 39: 1046–1055. 19266498 10.1002/eji.200838575

[30] Martin C, Al-Qaoud KM, Ungeheuer MN, Paehle K, Vuong PN, et al (2000) IL-5 is essential for vaccine-induced protection and for resolution of primary infection in murine filariasis. Med Microbiol Immunol 189: 67–74. 11138639 10.1007/pl00008258

[31] Martin C, Le Goff L, Ungeheuer MN, Vuong PN, Bain O (2000) Drastic reduction of a filarial infection in eosinophilic interleukin-5 transgenic mice. Infect Immun 68: 3651–3656. 10816524 10.1128/iai.68.6.3651-3656.2000 PMC97655

[32] Simons JE, Gray CA, Lawrence RA (2010) Absence of regulatory IL-10 enhances innate protection against filarial parasites by a neutrophil-independent mechanism. Parasite Immunol 32: 473–478. 20591117 10.1111/j.1365-3024.2010.01210.x

[33] Simons JE, Rothenberg ME, Lawrence RA (2005) Eotaxin-1-regulated eosinophils have a critical role in innate immunity against experimental Brugia malayi infection. Eur J Immunol 35: 189–197. 15593125 10.1002/eji.200425541

[34] Carter T, Sumiya M, Reilly K, Ahmed R, Sobieszczuk P, et al (2007) Mannose-binding lectin A-deficient mice have abrogated antigen-specific IgM responses and increased susceptibility to a nematode infection. J Immunol 178: 5116–5123. 17404294 10.4049/jimmunol.178.8.5116

[35] Gray CA, Lawrence RA (2002) A role for antibody and Fc receptor in the clearance of Brugia malayi microfilariae. Eur J Immunol 32: 1114–1120. 11920579 10.1002/1521-4141(200204)32:4<1114::AID-IMMU1114>3.0.CO;2-B

[36] Al-Qaoud KM, Fleischer B, Hoerauf A (1998) The Xid defect imparts susceptibility to experimental murine filariosis—association with a lack of antibody and IL-10 production by B cells in response to phosphorylcholine. Int Immunol 10: 17–25. 9488152 10.1093/intimm/10.1.17

[37] Hoffmann WH, Pfaff AW, Schulz-Key H, Soboslay PT (2001) Determinants for resistance and susceptibility to microfilaraemia in Litomosoides sigmodontis filariasis. Parasitology 122: 641–649. 11444617 10.1017/s0031182001007892

[38] Bouchery T, Ehrhardt K, Lefoulon E, Hoffmann W, Bain O, et al (2012) Differential tissular distribution of Litomosoides sigmodontis microfilariae between microfilaremic and amicrofilaremic mice following experimental infection. Parasite 19: 351–358. 23193519 10.1051/parasite/2012194351 PMC3671463

[39] Hawking F (1962) The role of the spleen in controlling the number of microfilariae (Dirofilaria immitis, D. repens, Litomosoides carinii and Dipetalonema witei) in the blood. Ann Trop Med Parasitol 56: 168–172. 13905508 10.1080/00034983.1962.11686104

[40] Haas B, Wenk P (1981) Elimination of microfilariae (Litomosoides carinii Filarioidea) in the patent and in the immunized cotton-rat. Trans R Soc Trop Med Hyg 75: 143–144. 7268849 10.1016/0035-9203(81)90046-8

[41] Wenk P, Kellermann E, Seeger V (1993) Turnover of microfilariae in small mammals. 1. Disintegration of microfilariae (Litomosoides sigmodontis) (Filarioidea: Nematoda) after intravenous injection into Sigmodon hispidus, the cotton rat. Trop Med Parasitol 44: 299–304. 8134771

