# Supplementary figures and images for: ST2 Deficiency Does Not Impair Type 2 Immune Responses during Chronic Filarial Infection but Leads to an Increased Microfilaremia Due to an Impaired Splenic Microfilarial Clearance
Source: PLoS One. 2014 Mar 24;9(3):e93072. doi: 10.1371/journal.pone.0093072 (PMC3963995; doi:10.1371/journal.pone.0093072)

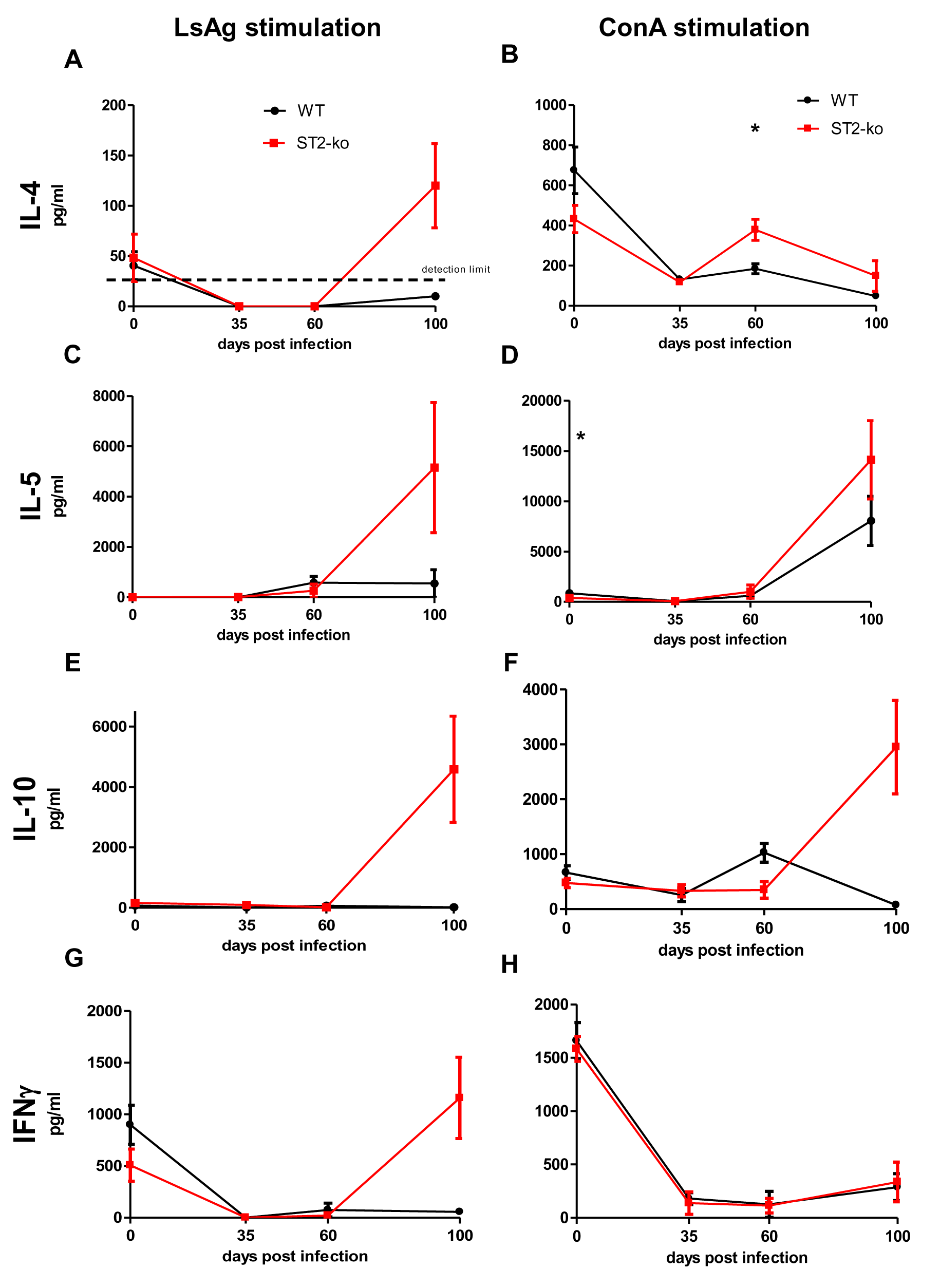

Supplement: Figure S1 — Lack of ST2 does not impair splenic Th2 cytokine production during L. sigmodontis infection. Isolated cells from the spleens of individual L. sigmodontis infected wild type (WT) and ST2-ko mice were cultured in vitro with either L. sigmodontis antigen (LsAg, left panel) or ConA (right panel). IL-4 (A, B), IL-5 (C, D), IL-10 (E, F), and IFNγ (G, H) within the cell culture supernatants were measured before infection (day 0) and on days 35, 60 or 100 post infection. Data is representative for two independent experiments for each measured time point with at least 5 mice per group. Differences were tested for statistical significance by Mann-Whitney-U-test, *p<0.05. (TIF) [file pone.0093072.s001.tif]

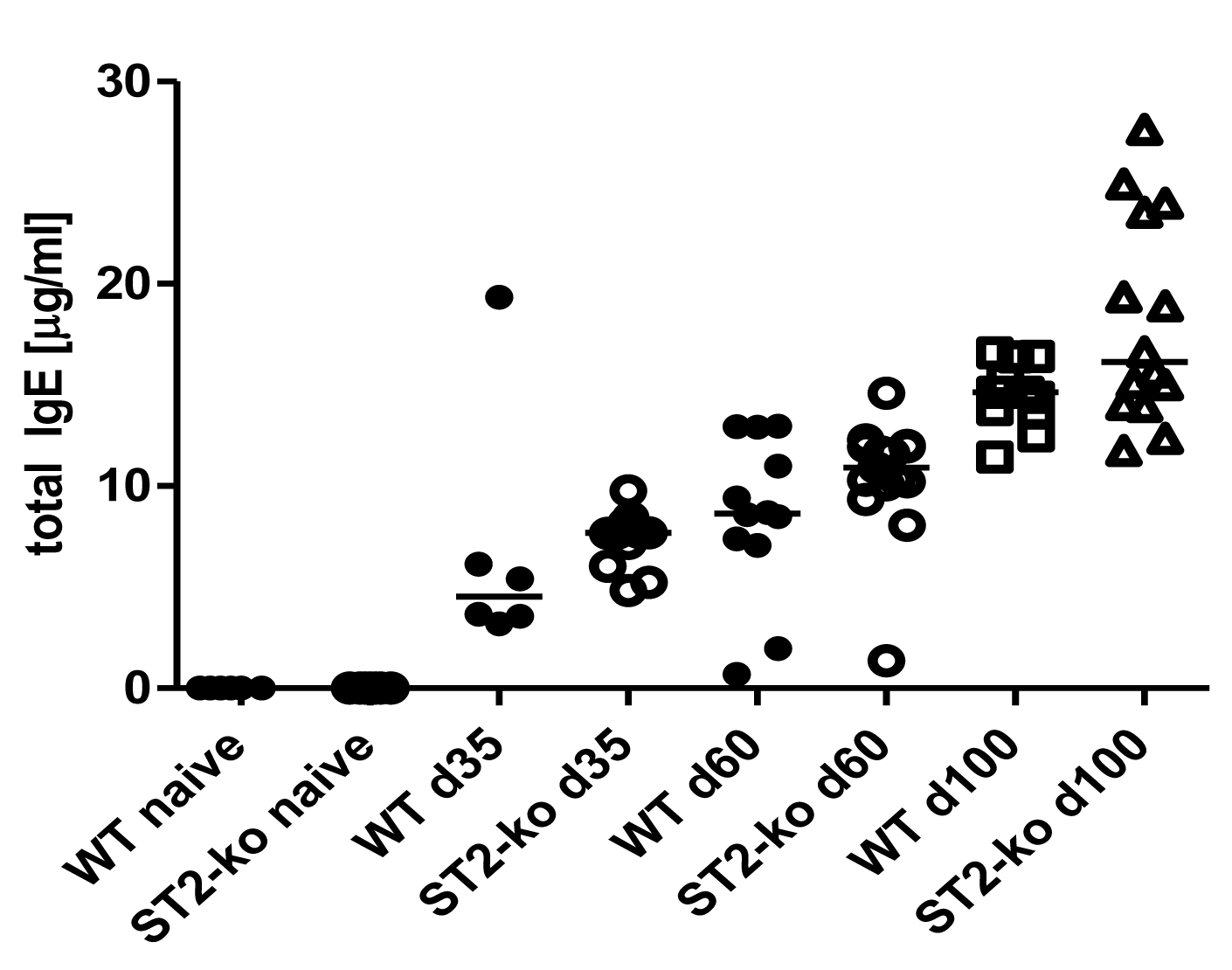

Supplement: Figure S2 — No differences in the production of total IgE between ST2-ko mice and wild type controls during L. sigmodontis infection. Total IgE antibody levels in plasma of ST2-ko mice and wild type (WT) controls on 35, 60 and 100 days post L. sigmodontis infection as well as naïve animals. Differences were tested for statistical significance by Mann-Whitney-U-test. (TIF) [file pone.0093072.s002.tif]

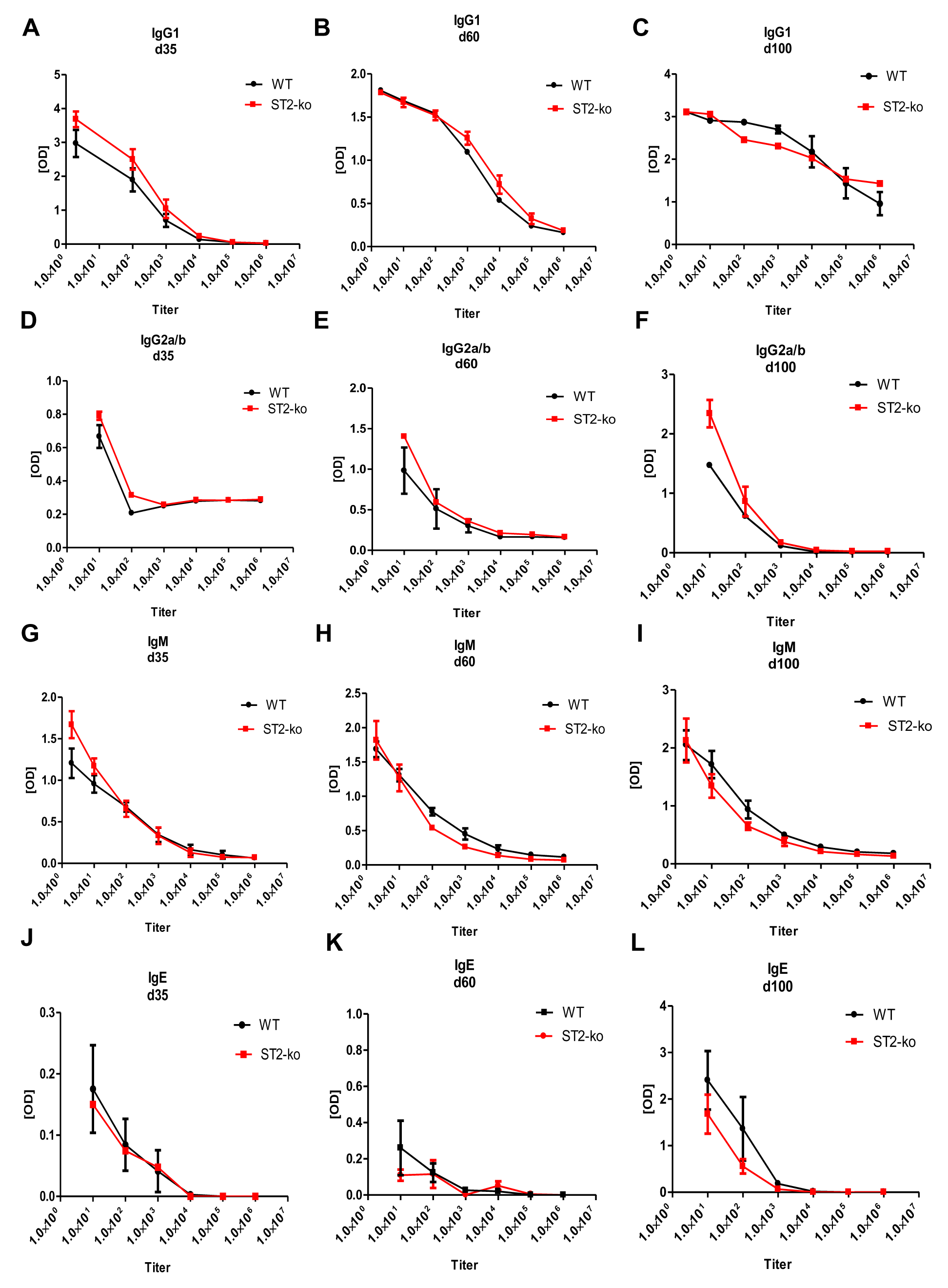

Supplement: Figure S3 — No differences in the production of filarial specific antibodies between ST2-ko mice and wild type controls. Optical density (OD) of IgG1 (A–C), IgG2a/b (D–F), IgM (G–I) and IgE (J–L) in plasma of ST2-ko mice and wild type (WT) controls 35, 60 and 100 days post L. sigmodontis infection. Differences were tested for statistical significance by Mann-Whitney-U-test. (TIF) [file pone.0093072.s003.tif]

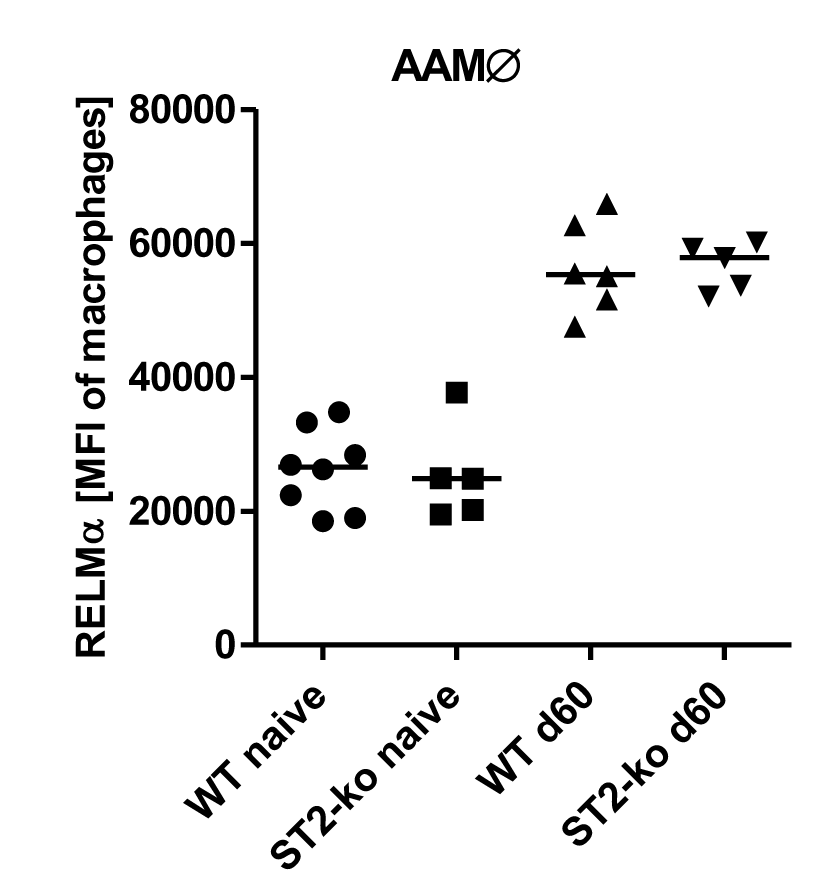

Supplement: Figure S4 — Similar expression of RELMα on thoracic cavity macrophages of ST2-ko mice and wild type controls. RELMα mean fluorescence intensity (MFI) from macrophages of naïve and 60 day L. sigmodontis infected wild type (WT) and ST2-ko mice. (TIF) [file pone.0093072.s004.tif]
